# In Patients With Obesity, the Number of Adipose Tissue Mast Cells Is Significantly Lower in Subjects With Type 2 Diabetes

**DOI:** 10.3389/fimmu.2021.664576

**Published:** 2021-05-21

**Authors:** David Lopez-Perez, Anaïs Redruello-Romero, Jesús Garcia-Rubio, Carlos Arana, Luis A. Garcia-Escudero, Francisco Tamayo, Jose D. Puentes-Pardo, Sara Moreno-SanJuan, Javier Salmeron, Armando Blanco, Julio Galvez, Josefa Leon, Ángel Carazo

**Affiliations:** ^1^ Department of Pharmacology, Faculty of Pharmacy, University of Granada, Granada, Spain; ^2^ Research Unit, Instituto de Investigación Biosanitaria de Granada (ibs.GRANADA), Granada, Spain; ^3^ Surgery Unit, San Cecilio University Hospital, Granada, Spain; ^4^ Endocrinology and Nutrition Unit, Virgen de las Nieves University Hospital, Granada, Spain; ^5^ Department of Statistics and Operative Research, Faculty of Sciences, University of Valladolid, Valladolid, Spain; ^6^ Cytometry and Microscopy Research Service, Instituto de Investigación Biosanitaria de Granada (ibs.GRANADA), Granada, Spain; ^7^ Gastroenterology Unit, San Cecilio University Hospital, Granada, Spain; ^8^ Department of Computer Science and Artificial Intelligence, University of Granada, Granada, Spain; ^9^ Centro de Investigación Biomédica En Red para Enfermedades Hepáticas y Digestivas (CIBER-EHD), Center for Biomedical Research, University of Granada, Granada, Spain; ^10^ Clinical Management Unit of Digestive Disease, San Cecilio University Hospital, Granada, Spain

**Keywords:** mast cell, T2D, adipose tissue, obesity, flow cytometry, angiogenesis, inflammation, adipogenesis

## Abstract

Type 2 diabetes (T2D) is a rising global health problem mainly caused by obesity and a sedentary lifestyle. In healthy individuals, white adipose tissue (WAT) has a relevant homeostatic role in glucose metabolism, energy storage, and endocrine signaling. Mast cells contribute to these functions promoting WAT angiogenesis and adipogenesis. In patients with T2D, inflammation dramatically impacts WAT functioning, which results in the recruitment of several leukocytes, including monocytes, that enhance this inflammation. Accordingly, the macrophages population rises as the WAT inflammation increases during the T2D status worsening. Since mast cell progenitors cannot arrive at WAT, the amount of WAT mast cells depends on how the new microenvironment affects progenitor and differentiated mast cells. Here, we employed a flow cytometry-based approach to analyze the number of mast cells from omental white adipose tissue (o-WAT) and subcutaneous white adipose tissue (s-WAT) in a cohort of 100 patients with obesity. Additionally, we measured the number of mast cell progenitors in a subcohort of 15 patients. The cohort was divided in three groups: non-T2D, pre-T2D, and T2D. Importantly, patients with T2D have a mild condition (HbA1c <7%). The number of mast cells and mast cell progenitors was lower in patients with T2D in both o-WAT and s-WAT in comparison to subjects from the pre-T2D and non-T2D groups. In the case of mast cells in o-WAT, there were statistically significant differences between non-T2D and T2D groups (p = 0.0031), together with pre-T2D and T2D groups (p=0.0097). However, in s-WAT, the differences are only between non-T2D and T2D groups (p=0.047). These differences have been obtained with patients with a mild T2D condition. Therefore, little changes in T2D status have a huge impact on the number of mast cells in WAT, especially in o-WAT. Due to the importance of mast cells in WAT physiology, their decrease can reduce the capacity of WAT, especially o-WAT, to store lipids and cause hypoxic cell deaths that will trigger inflammation.

## Introduction

The incidence of diabetes has increased four-fold in the last three decades, becoming the ninth death cause worldwide (1) and will affect 693 million people by 2045 (2). Of all the cases of patients with diabetes, type 2 diabetes (T2D) accounts for 90% (1). The development of T2D is strongly associated with obesity, which is driven by poor dietary habits and a sedentary lifestyle (1, 2).

When the caloric intake exceeds the energetic demands, adipocytes in white adipose tissue (WAT) store the excess energy as lipids, mainly triglycerides. Thus, when this positive energy imbalance continues for a long time, the WAT has to expand. So, adipocytes extend WAT *via* hypertrophy or hyperplasia. Adipocyte hyperplasia promotes insulin sensitivity and tissue homeostasis (3, 4). Conversely, adipocyte hypertrophy enhances insulin resistance and inflammation (3, 4). Interestingly, there are differences in adipose tissue depending on the depot location, including gene expression, metabolic features, and adipokine secretion (5, 6).

Noteworthy, subcutaneous white adipose tissue (s-WAT) has a higher adipogenic capacity than omental white adipose tissue (o-WAT). Therefore, when o-WAT expands, the amount of free fatty acids in the tissue increases. These free fatty acids trigger lipotoxicity and inflammation, producing tissue malfunctioning (7, 8). Consequently, the expansion of o-WAT, but not s-WAT, is a risk factor for developing T2D and cardiometabolic diseases (6, 9).

Mast cells are tissue-resident leukocytes located in all vascularized tissues. Depending on their location, they can develop two different phenotypes and become either connective tissue or mucosal mast cells (10, 11). Despite their widely known role in inflammation, mast cells also contribute to anti-inflammatory responses releasing TGF-beta and IL-10 (10, 12) and secreting proteases that can degrade pro-inflammatory cytokines (13). Additionally, they also participate in angiogenesis, lymphangiogenesis, tissue repair, and wound healing (12, 14). In these processes, it is crucial to remodel the extracellular matrix (ECM). Therefore, mast cells secrete proteases that directly digest the ECM or cause the proteolytic activation of ECM metalloproteases (13). In adipose tissue, mast cells have additional functions. They promote lipid uptake by macrophages and foam cell formation (15). In response to high glucose levels, mast cells release 15-deoxy-delta prostaglandin J2 (16, 17), which binds the peroxisome proliferator-activated receptor (PPAR) γ in pre-adipocytes promoting their differentiation to adipocytes (16, 17). Noteworthy, the absence of mast cells impairs the adipocyte differentiation (18).

When adipose tissue becomes insulin resistant, its physiology is altered. Firstly, it changes the adipokine profile (19). That affects mainly o-WAT since it secretes more pro-inflammatory adipokines than s-WAT (6). Secondly, some stress signals trigger the NLRP3 (NOD-, LRR- and pyrin domain-containing protein 3) inflammasome causing tissue inflammation (20, 21). These new conditions in the tissue microenvironment hamper adipogenesis (3, 4). This occurs by interfering with the PPAR-γ signaling pathway (22), which is critical for adipocyte hyperplasia (23, 24). The malfunctioning of the adipose tissue causes hyperglycemia and hyperlipidemia (24, 25). This leads to ectopic lipid accumulation in skeletal and heart muscle and several hepatic disorders, including hepatic steatosis (26–28).

Tissue-resident leukocytes, mainly macrophages and mast cells, play a key role in the homeostasis of the adipose tissue (8, 15, 16, 29). Nevertheless, the leukocytes recruited at WAT in patients with T2D mainly play a pro-inflammatory role (30, 31). Peripheral monocytes are recruited and differentiated to M1 macrophages that will produce more pro-inflammatory cytokines (7, 30). Such will recruit more monocytes forming a feed-forward loop (8, 29). Thus, as long as the glycemic control worsens, the number of macrophages sharply rises (7, 24, 29). However, the case of mast cells is not so straightforward. There is a tiny amount of mast cell progenitors circulating in peripheral blood (32). Noteworthy, these circulating mast cell progenitors arrive at some tissues, like the intestinal mucosa, but they do not enter in the WAT (33). Accordingly, several studies with murine models have demonstrated that the adipose tissue mast cell population depends on a local pool of embryonic mast cell progenitors instead of the circulating mast cell progenitors (11, 33, 34). Due to the scarce amount of circulating mast cell progenitors and their inability to reach adipose tissue, the number of mast cells will depend on how microenvironmental changes affect progenitor and differentiated mast cells.

## Materials and Methods

### Biochemical Parameters

Following the American Diabetes Association guidelines (35), T2D diagnosis requires two measures of fasting glucose. Accordingly, each patient gave 2 blood samples. The first one between 6 and 9 months before the surgery and it was used to measure the fasting plasma glucose. The second one was obtained on the day of the surgery before entering the operating room for a complete analysis. In both cases, the clinical analysis laboratory of San Cecilio University Hospital conducted the blood tests within 24h following approved protocols. Besides, fasting was defined as the absence of caloric intake for at least 8h.

For all patients, the Homeostatic Model Assessment for Insulin Resistance (HOMA-IR) was calculated with the data from the second blood test to evaluate insulin resistance.

### Cohort

This study includes 100 patients with morbid obesity who underwent laparoscopic bariatric surgery (gastric sleeve and gastric bypass). Patients with morbid obesity were further classified into non-T2D, pre-T2D, and T2D groups following the criteria of the American Diabetes Association (35). Patients with type I diabetes, gestational diabetes, genetic diabetes syndromes, diseases of the exocrine pancreas, drug-induced diabetes, and autoimmune diseases did not enter the cohort.

In addition to this, five patients without obesity, who underwent colonoscopy, provided samples of healthy mucosa. Such were employed as negative controls for the presence of a local pool of mast cell progenitors.

### Sample Processing

For each patient, two biopsies of adipose tissue were collected from laparoscopic bariatric surgery at San Cecilio University Hospital (Granada, Spain). The o-WAT (omental white adipose tissue) biopsies were taken from the greater omentum, close to the stomach. Alternatively, s-WAT (subcutaneous white adipose tissue) was sampled near the surgical incision. The biopsies were conserved in PBS and ice right after their extraction.

After that, visible blood vessels were removed from the samples. Then, approximately 2-2.5 g of each sample was weighed and cut into small pieces. Later, this was digested in RPMI 1640 medium supplemented with 2 mg/ml collagenase type I (Sigma) and 5 mM CaCl_2_ in a final volume of 10 ml, at 37°C during 2h. Subsequently, samples were washed with 35 ml of PBS, filtered through a 1 mm sieve, and centrifuged for 10 minutes at 900 x g. After, the pellet was resuspended in 10 ml of PBS, poured through a 100 μm filter, and centrifuged for 10 minutes at 900 x g. Finally, the pellet, that includes the stromal vascular fraction, was resuspended in 500 μl of antibody staining buffer (PBS, 2% fetal bovine serum, 0.09% albumin, and 0.05% sodium azide) and mixed with an internal standard (BD Truecount Absolute Counting Tubes) following manufacturer instructions.

The internal standard consists of a suspension of a known number of fluorescent microspheres, with size/complexity values far from any cellular population. The excitation and emission of these microspheres occur through a broad spectrum of wavelengths.

### Antibody Staining and Flow Cytometry

The stromal vascular fraction was labeled with 2 μl of controls or fluorophore-conjugated antibodies in Eppendorf tubes at room temperature for 20 minutes. After that, the cells were fixed, and the erythrocytes were lysed with 1 ml of BD FACS Lysing Solution for 30 minutes. Then, the samples were centrifuged 10 minutes at 3500 x g, and the pellets were resuspended in 500 μl of PBS. Subsequently, the samples were stored at 4°C until the next day. Flow cytometry was performed using a FACS ARIA III equipment, and data were acquired on a logarithmic scale. The internal standard was used to calculate the number of cells per mg of tissue.

**Figure 1:**
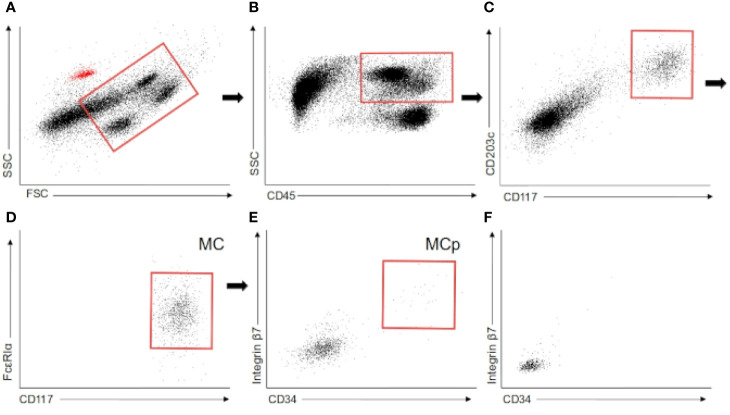
Flow cytometry. **(A–E)** Flow cytometry gating employed to identify mast cells and mast cell progenitors in white adipose tissue. The red dots in the first scatter plot are the autofluorescent beads employed in the quantification. **(F)** Mast cell progenitor gate in colonic mucosa. MC (mast cells), MCp (Mast cell progenitors).

The fluorescent-conjugated antibodies used to identify mast cells were: anti-CD45 PE-CF594 (clone HI30, BD), anti-CD117 APC (clone YB5.B8, BD), anti-FcϵRI PE-Cy7 (clone AER-37, BioLegend), and CD203c BV421 (clone NP4D6, BioLegend). Additionally, the antibodies CD34 BV785 (clone 561, BioLegend) and integrin β7 FITC (clone FIB504, BioLegend) were used in a subcohort of 15 patients (6 non-T2D, 6 pre-T2D, and 3 T2D). Compensation beads and isotype controls were purchased from BD Biosciences. MC were identified as CD45^+^ CD117^+^ CD203c^+^ FcϵRIα^+^ (**Figure 1A**).

### Statistical Analysis

The data of mast cells per g was log-transformed to promote symmetry and normality of the analyzed data. The Kolmogorov-Smirnov test was used to test the normality of the distribution of the data, and the Levene test was employed to check the homoscedasticity of the groups. To evaluate the differences between non-T2D, pre-T2D, and T2D groups the one-way ANOVA test was used followed by a Tukey HSD. Student’s t-test was used to analyze the differences between o-WAT and s-WAT in non-T2D, pre-T2D, and T2D groups independently. P-values below 0.05 were considered significant. Additionally, we employed the principal component analysis (PCA), the Linear Discriminant Analysis (LDA), and Random Forest Analysis to study the internal structure of our data and to find patterns and the most important variables to discriminate the three groups of patients. All the tests were conducted with R software (36).

## Results

### Cohort Baseline Characteristics

**Table 1** shows the average data of age, sex, hypertension, BMI, waist-hip index, insulin, glucose, HbA1c, HOMA-IR, triglycerides, cholesterol, LDL, and HDL. In this study, females account for 66% of the cohort, which may be due to social constraints in our geographic zone. Of note, the T2D group has a low HbA1c mean. The American Diabetes Association considers that the goal for patients with T2D is to have their HbA1c below 7% since it considerably reduces the risk for cardiovascular disease. Thus, our subjects with T2D have a mild condition (37).

**Table 1:** Cohort baseline characteristics.

	Non-T2D	Pre-T2D	T2D
**Number of patients**	41	32	27
**Age (years)**	43,5 ± 9.1	47.3 ± 11.8	50.5 ± 9.5
**Male/Female)**	16/25	11/21	7/20
**Hypertension (Yes/No)**	22/19	22/10	19/8
**Body Mass Index (kg/m^2^)**	45.2 ± 6.4	45.1 ± 6.9	43.0 ± 5.9
**Waist/Hip Index**	0.89 ± 0.08	0.93 ± 0.08	0.93 ± 0.09
**Insulin (units/ml)**	4.6 ± 2.8	7.8 ± 5.8	10.3 ± 6.1
**Glucose (mg/dl)**	87.7 ± 9.2	98.8 ± 14.9	154.9 ± 48.9
**HbA1c (%)**	5.3 ± 0.3	5.8 ± 0.4	6.6 ± 1.0
**HOMA-IR**	1.02 ± 0.68	1.95 ± 1.62	3.86 ± 2.57
**Triglycerides (mg/dl)**	138.0 ± 54.7	162.7 ± 68.0	149.7 ± 38.4
**Cholesterol (mg/dl)**	162.6 ± 38.0	151.6 ± 28.3	147.9 ± 45.0
**LDL (mg/dl)**	93.2 ± 32.2	83.3 ± 22.7	86.8 ± 40.5
**HDL (mg/dl)**	40.7 ± 10.9	35.5 ± 9.1	36.8 ± 10.0

The data comes from the big cohort (n=100). Data are expressed as mean ± standard deviation. T2D (type 2 diabetes).

### A Subpopulation of CD34+ Integrin ;4β7+ Mast Cells in Omental and Subcutaneous White Adipose Tissue but Not in Colonic Mucosa

Mast cells were identified in the whole cohort as CD45^+^ CD117^+^ CD203c^+^ FcϵRIα^+^ by flow cytometry (**Figures 1A–E**). Moreover, in the subcohort of 15 patients, a CD34^+^ integrin b7^+^ subpopulation of mast cells was analyzed. This subpopulation was found in both types of WAT in these 15 patients (**Figure 1E**). Oppositely, this subpopulation was absent in the five samples of colonic mucosa (**Figure 1F**). Previous studies have shown that this subpopulation is made of mast cell progenitors in murine peripheral tissues (38, 39) and human blood (32). Besides, it has been reported that, in humans, the heterogeneous pool of progenitor cells in WAT contains cells that can give rise to mast cells (34). Therefore, the expression of CD34 and integrin b7 in a small fraction of the mast cell pool strongly suggests the presence of a stable pool of progenitor cells committed with mast cell lineage in human WAT.

These mast cell progenitors are more abundant in o-WAT than in s-WAT and seem to decrease in the T2D group in both types of WAT (**Table 2**). The proportion of mast cell progenitors in the whole population is displayed in **Table 3**.

**Table 2:** Number of mast cell progenitors per gram of white adipose tissue.

	Non-T2D	Pre-T2D	T2D
**o-WAT**	1623.45 ± 3360.70	880.07 ± 1050.14	62.90 ± 24.55
**s-WAT**	596.23 ± 999.85	302.72 ± 145.38	60.2 ± 30.94

The data comes from the small cohort (n=15). Data are expressed as mean ± standard deviation. T2D, type 2 diabetes; o-WAT, omental white adipose tissue; s-WAT, subcutaneous white adipose tissue.

**Table 3:** Proportion of mast cell progenitors in the whole mast cell pool.

	Non-T2D	Pre-T2D	T2D
**o-WAT**	2.54% ± 4.29	3.04% ± 2.54	0.93% ± 0.75
**s-WAT**	2.47% ± 1.79	2.04% ± 1.56	0.57% ± 0.27

The data comes from the small cohort (n=15). Data are expressed as mean ± standard deviation. T2D, type 2 diabetes; o-WAT, omental white adipose tissue; s-WAT, subcutaneous white adipose tissue.

### The Number of Mast Cells in Omental and Subcutaneous White Adipose Tissue Decrease in Patients With T2D

**Figure 2:**
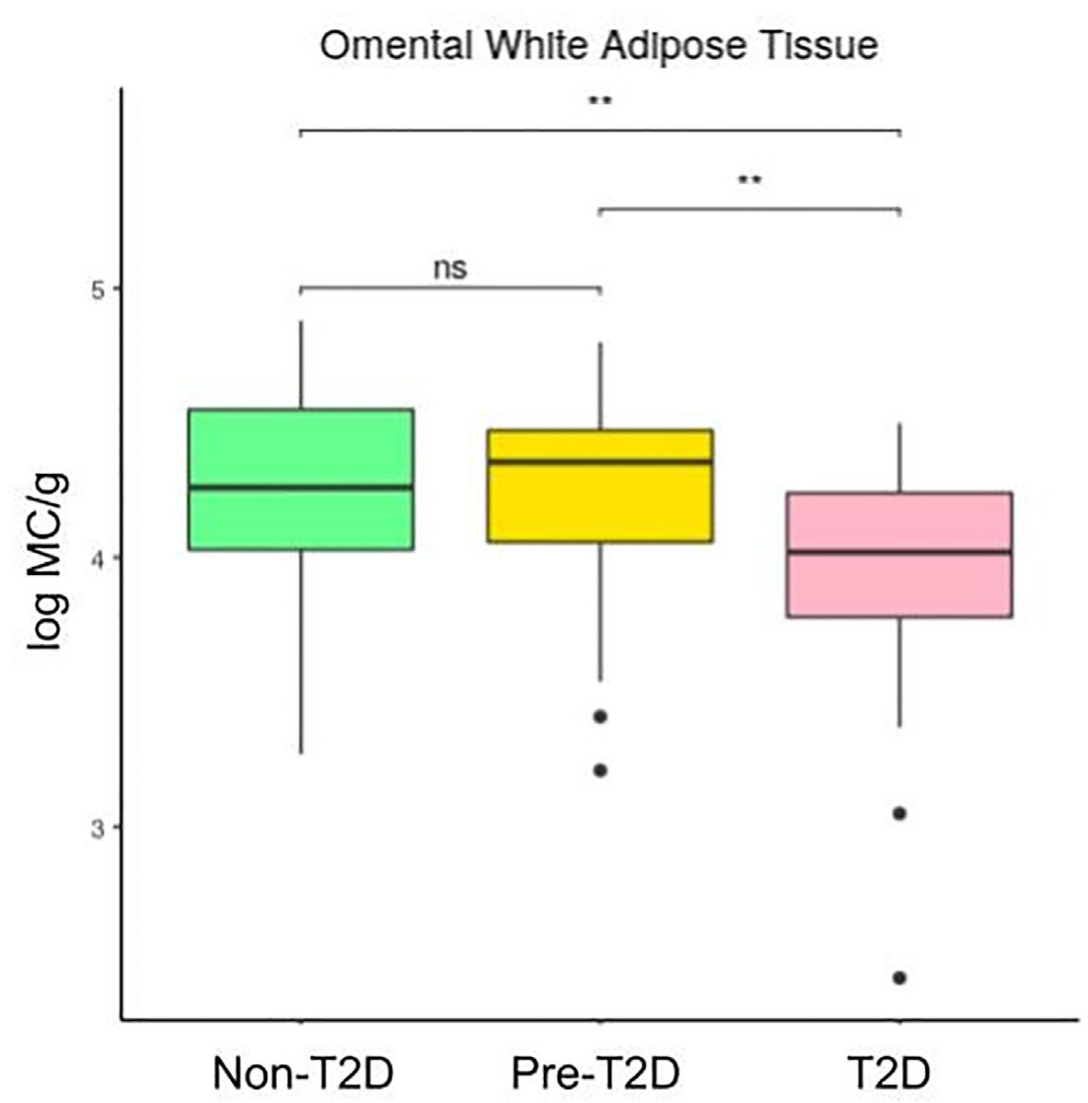
Differences in mast cells per gram of tissue in omental white adipose tissue depending on the type 2 diabetes status. The data comes from the big cohort (n = 100). MC (mast cells), T2D (type 2 diabetes), “ns” (p-value > 0.05), “ ** ” (0,01 > p-value).

**Figure 3:**
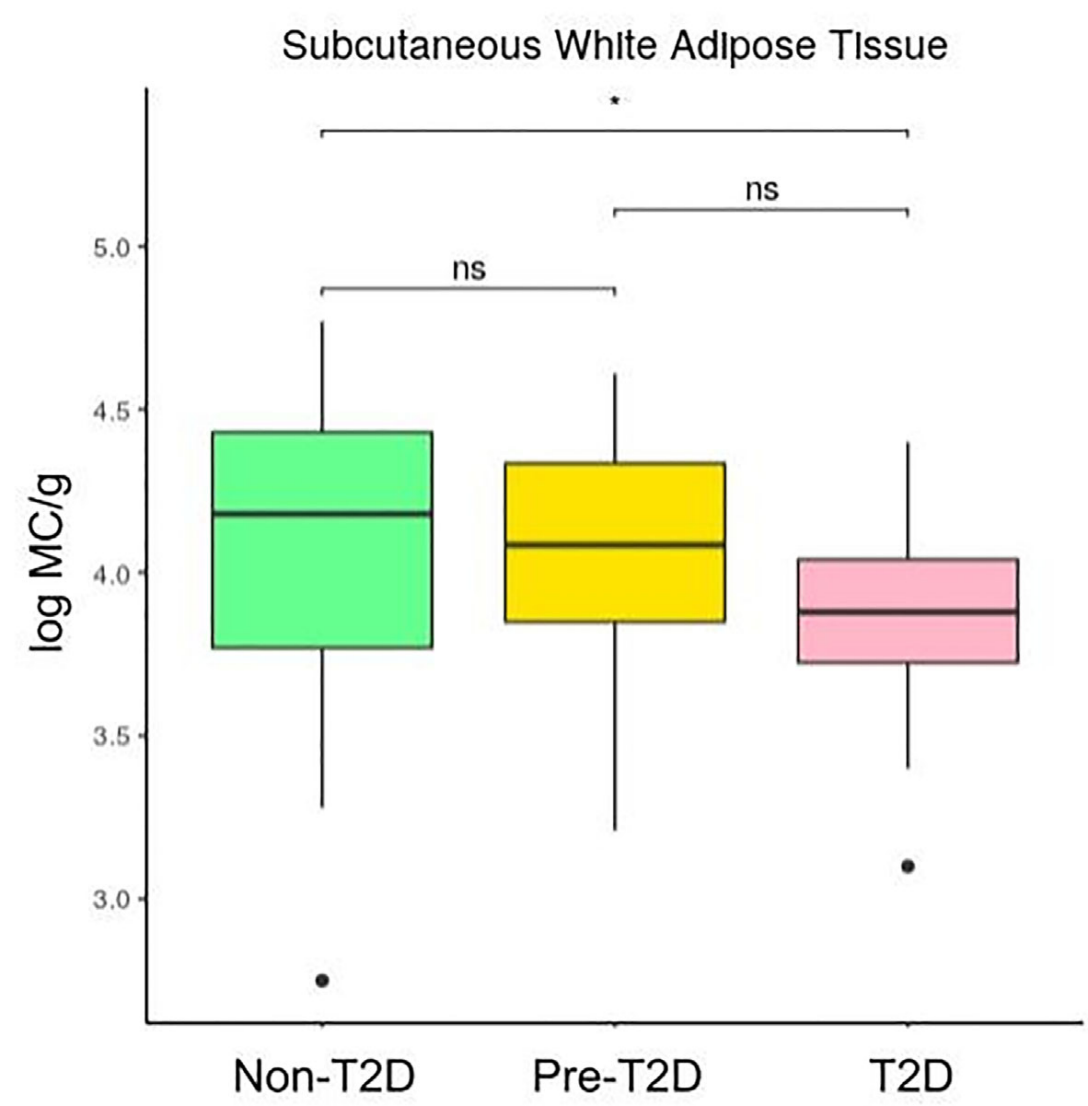
Differences in mast cells per gram of tissue in subcutaneous white adipose tissue depending on the type 2 diabetes status. The data comes from the big cohort (n = 100). MC (mast cells), T2D (type 2 diabetes), “ns” (p-value > 0.05), “ * ” (0.05 > p-value > 0.01).

**Figures 2** and **3** show that the number of mast cells was smaller in the T2D group in both o-WAT and s-WAT. In the case of o-WAT (**Figure 2** and **Supplementary Figure 1**), the differences are between non-T2D and T2D groups (p=0.0031), as well as pre-T2D and T2D groups (p=0.0097). However, in s-WAT (**Figure 3** and **Supplementary Figure 2**), the differences are only between non-T2D and T2D groups (p=0.047). These results indicate that mast cells of both o-WAT and s-WAT are negatively affected by the dysregulation of glucose metabolism, particularly when T2D is reached.

### There Are Differences in the Number of Mast Cells Between Omental and Subcutaneous White Adipose Tissue in Non-T2D and Pre-T2D Groups but Not in the T2D Group

**Figure 4:**
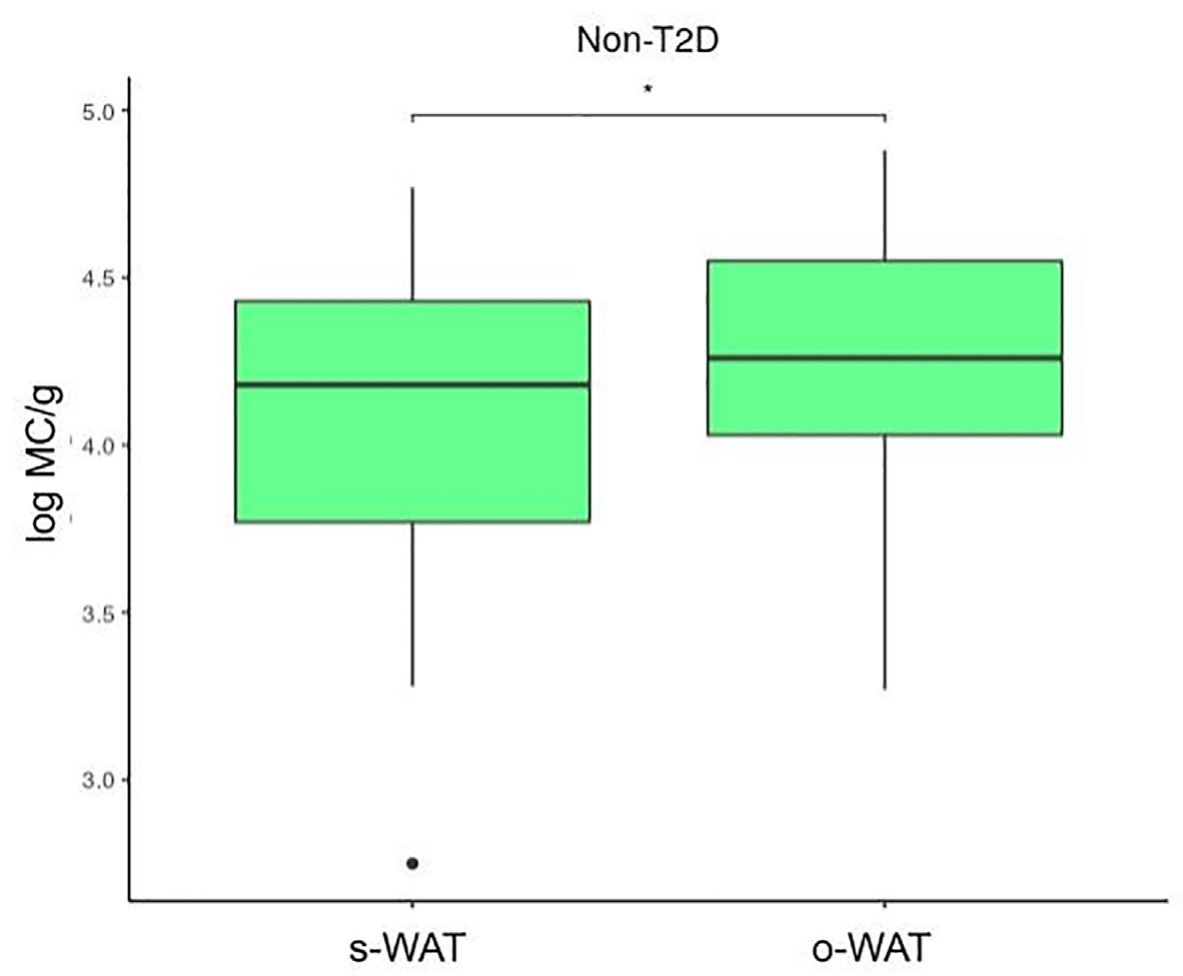
Differences in mast cells per gram of tissue between subcutaneous and omental white adipose tissue in patients without type 2 diabetes. The data comes from the big cohort (n = 100). MC (mast cells), s-WAT (subcutaneous white adipose tissue), o-WAT (omental white adipose tissue), T2D (type 2 diabetes), “ * ” (0.05 > p-value > 0.01).

**Figure 5:**
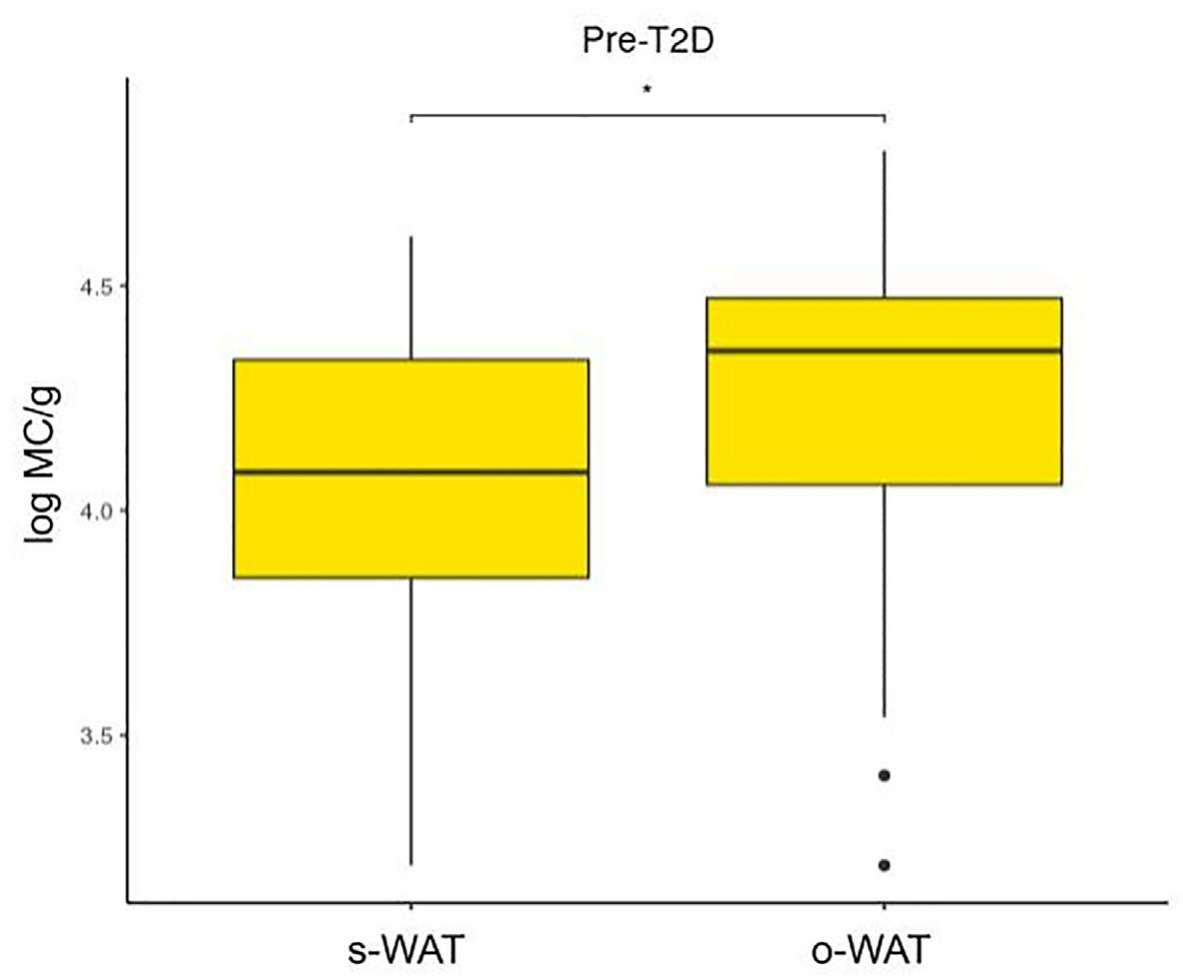
Differences in mast cells per gram of tissue between subcutaneous and omental white adipose tissue in patients with pre-type 2 diabetes. The data comes from the big cohort (n = 100). MC (mast cells), s-WAT (subcutaneous white adipose tissue), o-WAT (omental white adipose tissue), T2D (type 2 diabetes), “ * ” (0.05 > p-value > 0.01).

**Figure 6:**
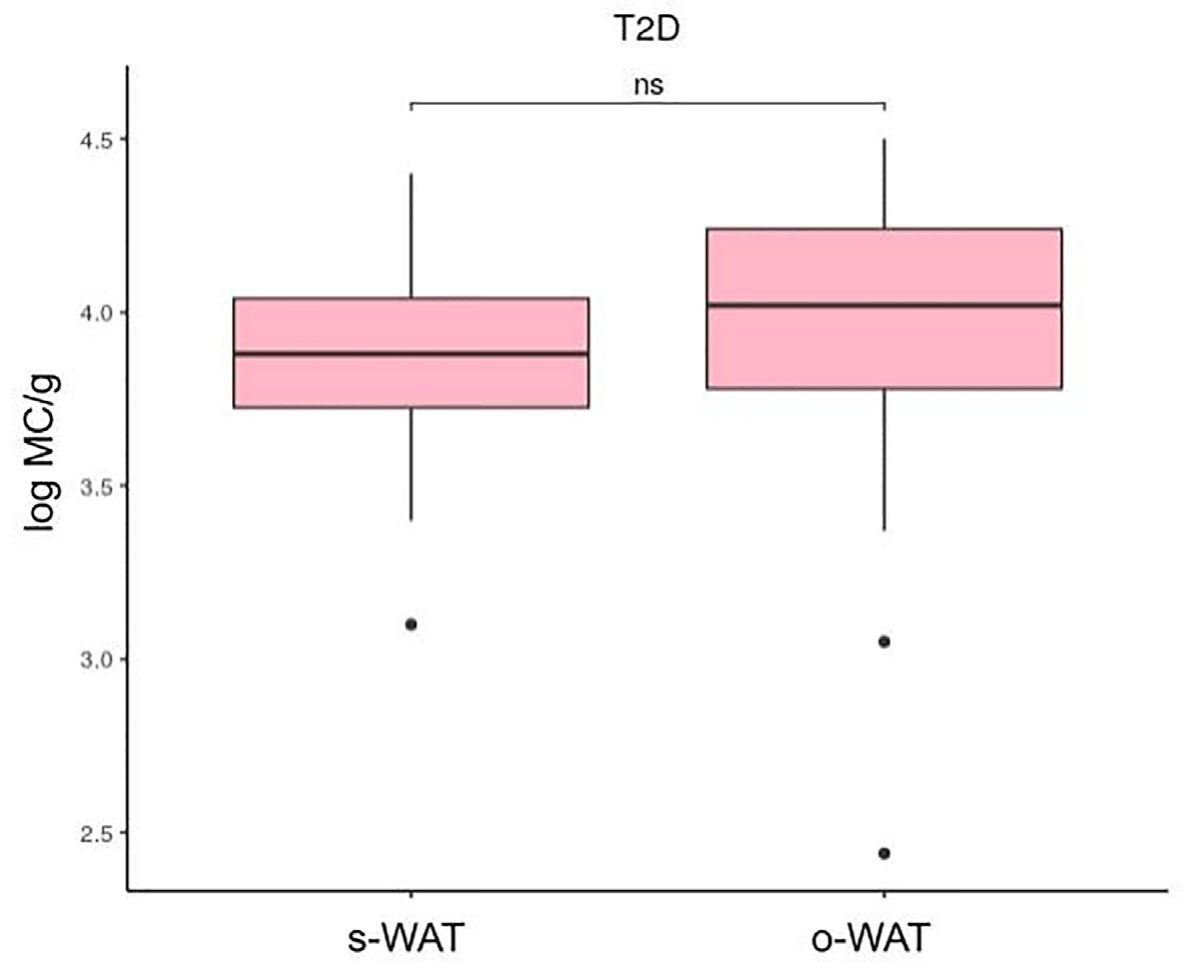
Differences in mast cells per gram of tissue between subcutaneous and omental white adipose tissue in patients with type 2 diabetes. The data comes from the big cohort (n = 100). MC (mast cells), s-WAT (subcutaneous white adipose tissue), o-WAT (omental white adipose tissue), T2D (type 2 diabetes), “ns” (p-value > 0.05).

**Figures 4**, **5**, and **6** show that the difference in the number of mast cells between o-WAT and s-WAT becomes smaller in patients with T2D. Accordingly, these differences are significant only in non-T2D group (p=0.016) and pre-T2D group (p=0.021). Under normal conditions, the number of mast cells is higher in o-WAT than in s-WAT. Such occurs because o-WAT but not s-WAT is in contact with microbial products from the intestinal microbiota and plays a crucial role in peritoneal cavity immunology (40–42). Nonetheless, although in T2D group there is a sharp decrease in the number of mast cells in both locations, they are not equally affected. These results show that T2D condition has a higher impact on the number of mast cells in o-WAT than in s-WAT.

### The Number of Mast Cells Is a Good Predictor of T2D Status.

**Figure 7:**
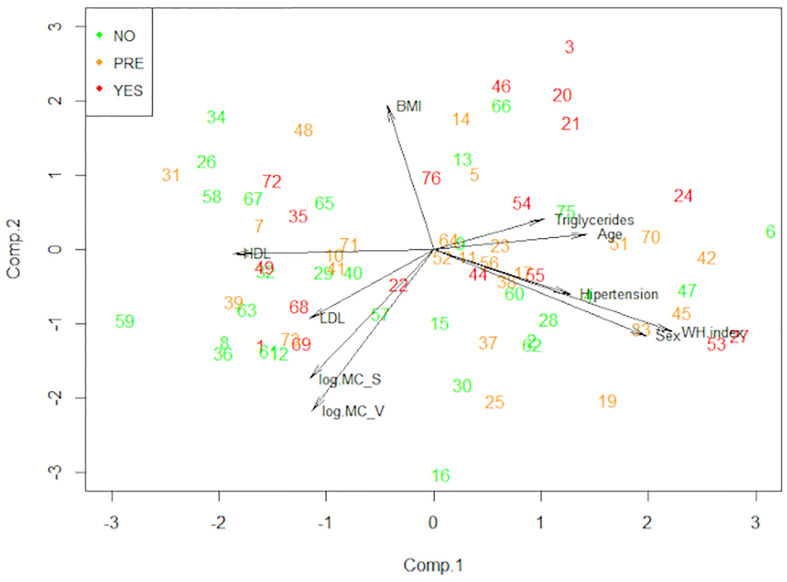
Principal component analysis (PCA). This technique allows us to observe the patterns of our data reducing its dimension. The data comes from the big cohort (n = 100). NO (patients without type 2 diabetes), PRE (patients with pre-type 2 diabetes), YES (patients with type 2 diabetes), BMI (Body Mass Index), MC_V (mast cells from omental white adipose tissue), MC_S (mast cells from subcutaneous white adipose tissue), WH index (waist hip index).

To see the patterns between our variables and the three groups of patients simplifying our high dimensional data, we performed a principal component analysis (**Figure 7**). This statistical analysis serves to summarize the variables dependence structure and to visualize the observed values. Interestingly, the non-T2D group tends to have higher numbers of mast cells in both o-WAT and s-WAT. The HbA1c was not included in this analysis and the following because it is used to define the groups.

**Figure 8:**
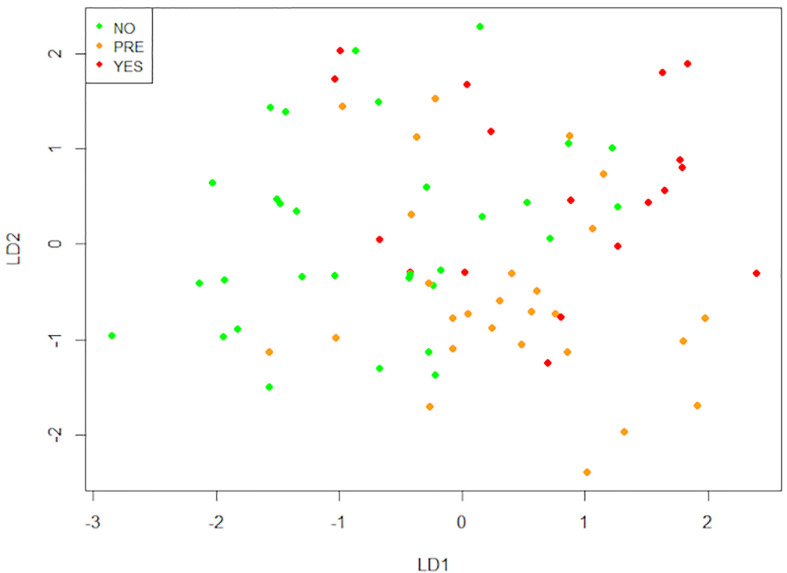
Linear discriminant analysis (LDA). This technique finds the linear combinations of variables that better separate the observations. The data comes from the big cohort (n = 100). NO (patients without type 2 diabetes), PRE (patients with pre-type 2 diabetes), YES (patients with type 2 diabetes), LD1 (linear discriminant 1), LD2 (linear discriminant 2).

After that, we performed a Linear Discriminant Analysis (LDA) to seek the linear combinations of the original variables that better serve to “separate” the observations in the transformed space when considering the levels of the “Type 2 Diabetes” variable (no, pre, yes) (**Figure 8** and **Supplementary Table 1**). We can see that the first linear combination (LD1) separates well those observations. Negative values of LD1 are associated with “No” values, while positive ones are more related to “Pre” and “Yes”. Later, we studied the correlations of each variable with LD1 (**Table 4**). The stronger correlations are with age (0.50), waist-hip index (0.43), and the number of mast cells in o-WAT (-0.38). The negative sign of the correlation between mast cells and LD1 indicates that the number of mast cells in o-WAT is higher in patients with low LD1 values. Since the patients in the non-T2D group tend to have negative values of LD1, this agrees with our previous results.

**Table 4:** Correlation of LD1 with the variables.

Variable	Correlation Coefficient
Age	0.502
Waist-hip index	0.434
MC (o-WAT)	-0.380
HDL	-0.350
LDL	-0.319
Hypertension	0.300
Triglycerides	0.289
Sex (Male)	-0.280
MC (s-WAT)	-0.082
BMI	0.063

The data comes from the big cohort (n=100). MC, mast cells; o-WAT, omental white adipose tissue; s-WAT, subcutaneous white adipose tissue.

**Figure 9:**
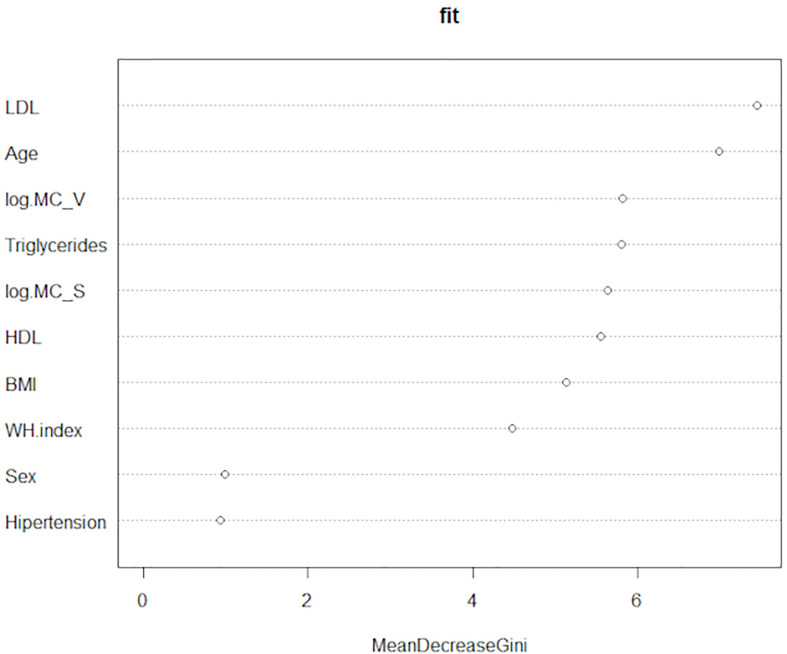
Random Forests Analysis. This technique measures the importance of the variables to discriminate the three levels of the categorical variable “Type 2 diabetes”. The data comes from the big cohort (n = 100). MC_V (mast cells in omental white adipose tissue), MC_S (mast cells in subcutaneous white adipose tissue), WH index (waist-hip index).

Finally, we performed the Random Forests Analysis. This technique also serves to measure the importance of the variables in discriminating the (three) levels of the categorical variable “Type 2 Diabetes”. That importance is determined through the “mean decrease in the Gini index”. Compellingly, the number of mast cells in o-WAT and s-WAT are respectively the third and fifth most important variables (**Figure 9** and **Supplementary Table 2**). They are above some variables frequently studied in patients with T2D, including HDL, BMI, and waist-hip index.

These results together indicate that mast cells in adipose tissue (especially o-WAT) have a strong relationship with T2D status. Considering this occurs with patients with mild T2D, this phenomenon is expected to be more prominent in patents with T2D and poor glycemic control.

## Discussion

Mast cells are widely known for their role in inflammation and allergy. Thus, at first sight, it seems plausible that they play a deleterious role in obesity and T2D. This statement was supported by studies about mast cell numbers and mast cell stabilization in adipose tissue, and knockout models. Firstly, a previous study reported an increase in the number of mast cells in adipose tissue as the glycemic control worsens (43). However, this study worked with histological slides and normalized the number of mast cells with the surface of fibrosis in the tissue biopsy. This work provides valuable information about the tissue structure, but the cell counting method is not precise. Besides, mast cells are present not only in fibrotic tissue but also surrounding blood vessels (15). Here, we have digested the whole sample (2-2.5g), and we have employed a more accurate cell-counting method. Secondly, other studies in murine models reported that cromolyn, a mast cell stabilizer, reduces obesity and adipose tissue fibrosis, while it promotes insulin sensitivity (44, 45). Even though cromolyn is a widely used drug to treat asthma (46), its mechanism of action is still poorly understood (47). Curiously, mucosal mast cells are sensitive to cromolyn, but connective tissue mast cells are not (47, 48). Also, TNFα production by cell extracts from the peritoneum shows no significant difference between wild-type and mast cell-deficient mice (47). Nevertheless, in both wild-type and mast cell-deficient mice, cromolyn inhibited TNFα production in a dose-dependent manner (47). Therefore, the current knowledge suggests that cromolyn has a positive effect on patients with T2D but acting in other cell types. Accordingly, cromolyn can inhibit neutrophils (49–51), eosinophils (49, 52), monocytes (49, 52), and macrophages (52). Finally, the genetic ablation of c-kit, a critical receptor for mast cell development, protected mice from weight gain and insulin resistance (44). Nonetheless, c-kit is also crucial for the development of other leukocyte lineages. So, further experiments demonstrated that c-kit ablation but not mast cell depletion is what improves the metabolic profile in mice (53). Although more genetic models explored the effect of the absence of mast cells in WAT, the current evidence is that it does not protect from obesity and insulin resistance (54, 55). In a nutshell, mast cells are not critical players driving adipose tissue inflammation in patients with T2D.

The BMI is similar regardless of the T2D status. Therefore, it suggests that the problem is not the expansion of the adipose tissue itself but the mechanism to achieve it. When adipocytes expand the adipose tissue in response to an increase in the nutrients available, oxygen diffusion drops. This mild hypoxia produces stress signals that promote angiogenesis (56). Of note, angiogenesis is impaired in patients with T2D (57). Thus, hypoxic stress becomes greater, and some cells die because of it. These dead cells in the hypoxic areas of the tissue trigger inflammation and fibrosis (3, 4, 7, 21, 56).

Mast cells sense this hypoxic condition *via* reactive oxygen species (ROS) (15) and hypoxia-inducible factor 1α (HIF1α) production (58). Then, to promote angiogenesis, they release pro-angiogenic factors (VEGF, bFGF, TGF-beta), proteases to remodel the ECM, histamine to increase vascular permeability, and heparin (15). Moreover, mast cells downregulate the production of pro-inflammatory cytokines in response to hypoxic conditions (58). Apart from this, mast cells interact with endothelial cells to promote their proliferation (15) and the release of angiogenic factors (59). Consequently, in the absence of mast cells, the adipose tissue is less vascularized (60).

Furthermore, mast cells also contribute to foam cell formation (15) and pre-adipocyte to adipocyte differentiation (16–18). Therefore, the mast cell population reduction will affect the adipose tissue capacity to uptake and store lipids. Thus, the amount of free fatty acids will increase, causing hyperlipidemia. Such activates TLR2 and TLR4 on macrophages promoting inflammation (4, 21). Also, it boosts gluconeogenesis in the liver, prompting hyperglycemia (61) and causing hepatic steatosis (8). Finally, hyperlipidemia is an indicator of poor prognosis for the development of cardiovascular diseases (62).

Unfortunately, there are very few studies about mast cells in WAT, and most of them employ murine models. So, little is known about mast cells in human WAT. Moreover, many of the studies about mast cells in humans use histological slides instead of flow cytometry. Besides, the differences between humans and mice can also affect.

In mice, fibrosis in WAT causes tissue dysfunction. However, the situation in humans is more complicated. Fibrosis in human s-WAT was associated with a pathological metabolic profile, bigger adipocytes, and low weight loss after bariatric surgery (63). Inversely, fibrosis in o-WAT is associated with smaller adipocytes (64, 65). Since fibrosis in o-WAT limits adipocyte hypertrophy, it provides a better metabolic profile (64, 65). Mast cells locate in large numbers in fibrotic tissue. Where they can produce or degrade collagen depending on the molecular context (66). In adipose tissue fibrosis, it is unknown the role they play (66). Nonetheless, in the liver, mast cells play an antifibrotic role (67).

Previously, we reported three key aspects about the changes in adipose tissue physiology after weight loss induced by bariatric surgery (68). Firstly, WAT undergoes extensive tissue remodeling that increases insulin sensitivity. Secondly, pre-adipocytes increase their number. Thirdly, the size of adipocytes decreases. These three changes strongly suggest a remodeling process in WAT that leads to glucose metabolism normalization. Furthermore, bariatric surgery reduces the inflammatory status of both o-WAT and s-WAT, including a sharp decrease in the number of neutrophils (68). Importantly, after bariatric surgery, the number of mast cells increases 10-fold in o-WAT and 4-fold in s-WAT (68). Therefore, since mast cells increase in number (68) and promote adipogenesis (16–18), they may play a major role in the remodeling of the adipose tissue.

The study of Goldstein et al. (69) showed that in patients with obesity, a higher number of mast cells in o-WAT is associated with a lower cardiometabolic risk. Moreover, a higher amount of mast cells in o-WAT is linked with a higher weight loss after bariatric surgery. Finally, higher number of mast cells in o-WAT was associated with higher insulin sensitivity in hepatocytes (69).

Mast cells have a close relationship with WAT and have an essential function in its physiology (15–18, 58). Mast cell progenitors arrive at WAT during the embryonic stage (33). After that, the whole differentiation of mast cells takes place in WAT under the influence of the local microenvironment (70, 71). Nevertheless, currently, the differences between bone marrow progenitors and WAT progenitors are poorly understood.

Mast cells contribute to WAT homeostasis interacting with several adipokines including leptin (72) and lipoproteins like LDL (73) and stromal cells (74). Moreover, in cold conditions, the number of mast cells increases in WAT. These mast cells promote the browning of WAT in response to norepinephrine (55, 75, 76).

In conclusion, the number of mast cells decreases in patients with T2D. Noteworthy, the HbA1c mean difference between groups is small. Therefore, little changes in glycemia have a huge impact on the number of mast cells in WAT, especially in o-WAT. Since mast cells play a prominent homeostatic role in adipose tissue, their decrease can contribute to the deterioration of patients with T2D. One of the limitations of this study is that the patients with T2D have a mild condition (HbA1c <7%). It would be interesting to include a group with severe T2D. However, patients with severe T2D have an increased risk of complications during surgery and usually follow other therapeutic strategies.

## Data Availability Statement

The raw data supporting the conclusions of this article will be made available by the authors, without undue reservation.

## Ethics Statement

The studies involving human participants were reviewed and approved by Andalucia’s Biomedical Research Ethics Committee of Granada (CEIM/CEI Granada). The patients/participants provided their written informed consent to participate in this study.

## Author Contributions

JG-R and FT performed the surgeries. DL-P, AR-R, JP-P, and SM-S performed experiments. DL-P, LG-E, AB, and CA analyzed and interpreted data. DL-P, CA, JS, JG, JL, and ÁC interpreted data, drafted the manuscript, and contributed with intellectual content. JG, JL, and ÁC designed and supervised the study. All authors contributed to the article and approved the submitted version.

## Funding

Instituto de Salud Carlos III (grant PI18/01947). Instituto de Salud Carlos III is a research institution from the Spanish government that funds biomedical research. Instituto de Salud Carlos III funded all the experiments.

## Conflict of Interest

The authors declare that the research was conducted in the absence of any commercial or financial relationships that could be construed as a potential conflict of interest.
