# Supplementary material for: In Patients With Obesity, the Number of Adipose Tissue Mast Cells Is Significantly Lower in Subjects With Type 2 Diabetes
Source: Front Immunol. 2021 May 21;12:664576. doi: 10.3389/fimmu.2021.664576 (PMC8177010; doi:10.3389/fimmu.2021.664576)

***Supplementary Material***

# Supplementary Tables

**Supplementary Table 1**. Coefficients of the linear discriminants LD1 and LD2. The data comes from the big cohort (n=100). MC (Mast cells), o-WAT (omental white adipose tissue), s-WAT (subcutaneous white adipose tissue), WH index (waist-hip index).

| **Variable** | **LD1** | **LD2** |
| --- | --- | --- |
| Age | 0.232 | -0.075 |
| Sex (Male) | -2.314 | -0.601 |
| Hipertension (Yes) | 0.454 | -0.667 |
| BMI | 0.059 | -0.866 |
| WH index | 0.876 | 0.042 |
| Triglycerides | 0.022 | -0.433 |
| HDL | -0.460 | 0.202 |
| LDL | -0.189 | 0.121 |
| Log MC (o-WAT) | 0.227 | -0.853 |
| Log MC (s-WAT) | 0.075 | -0.047 |

**Supplementary Table 2**. Mean decrease in the Gini Index in the Random Forests analysis. The data comes from the big cohort (n=100). MC (Mast cells), o-WAT (omental white adipose tissue), s-WAT (subcutaneous white adipose tissue), WH index (waist-hip index).

| **Variable** | **Mean decrease in the Gini Index** |
| --- | --- |
| LDL | 7.441 |
| Age | 6.986 |
| Log MC (o-WAT) | 5.808 |
| Triglycerides | 5.796 |
| Log MC (s-WAT) | 5.639 |
| HDL | 5.555 |
| BMI | 5.137 |
| WH index | 4.473 |
| Sex | 0.996 |
| Hipertension | 0.941 |

## 2 Supplementary Figures

Supplementary Figure 1. Tukey HSD plot for the confidence interval of pairwise comparisons of the mean number of mast cells per gram of tissue in omental white adipose tissue. The data comes from the big cohort (n=100). T2D (type 2 diabetes).
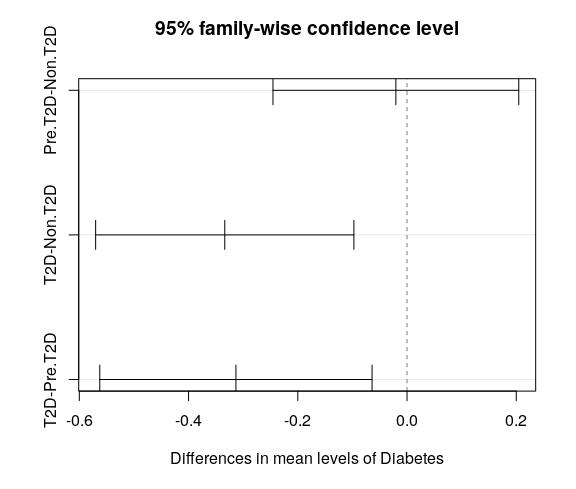


Supplementary Figure 2. Tukey HSD plot for the pairwise comparison of the mean number of mast cells per gram of tissue in subcutaneous white adipose tissue. The data comes from the big cohort (n=100). T2D (type 2 diabetes).
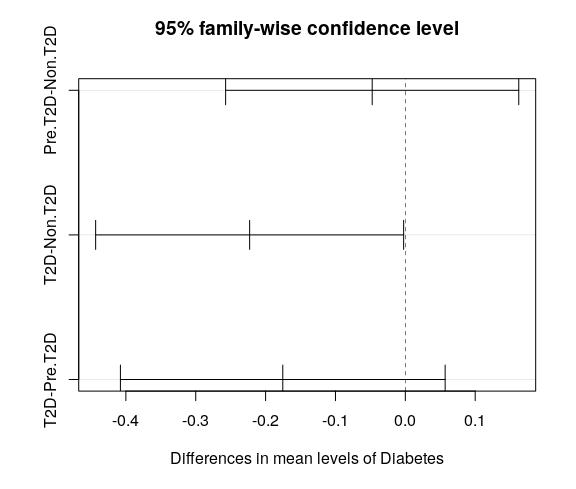

Supplement: Supplementary file 1 [file DataSheet_2.docx]
